# Mineralogical, geochemical, and geotechnical features of lateritic soils from termite mounds in two contrasting savannah areas (central Cameroon) as raw materials for brick making

**DOI:** 10.1016/j.heliyon.2023.e17257

**Published:** 2023-06-14

**Authors:** Jean-Marc Kessoum Adamou, Roger Firmin Donald Ntouala, Estelle Ndome Effoudou, Marie Thérèse Nanga Bineli, Arnaud Ngo'o Ze, Gouban Hamadjida, Vincent Laurent Onana

**Affiliations:** a Department of Earth Sciences, Faculty of Science, University of Yaoundé I, P.O. Box, 812, Yaoundé, Cameroon; b Department of Mining Engineering and Mineral Processing, National Advanced School of Mines and Petroleum Industries, University of Maroua, P.O. Box 08, Kaélé, Cameroon; c Higher Teacher Training College, University of Yaoundé I, P.O. Box 47, Yaoundé, Cameroon; d National Institute of Cartography, P.O. Box 157, Yaoundé, Cameroon

**Keywords:** Termite mound soils, Characterization, Compressed earth bricks, Mechanical properties, Fired clay bricks, Centre Cameroon

## Abstract

Termite mound soils (TMS) from humid savannah (HS) and dry savannah (DS) were evaluated as raw materials for compressed earth bricks (CEB) and fired bricks. Mineralogy and major elements geochemistry were performed by X-Ray Diffraction and X-Ray Fluorescence, respectively. Physico-mechanical characteristics of unfired and fired bricks at 900, 950, 1000, 1050, and 1100 °C after 7 curing days were evaluated. The studied TMS are made up of quartz, muscovite, anatase, kaolinite, hematite, and goethite. Illite is present in humid savannah while in DS gibbsite appears. These materials are rich in SiO_2_ (58.96–61.79 wt%), Al_2_O_3_ (16.93–18.78 wt%), and Fe_2_O_3_ (7.41–10.33 wt%). The TMS from both HS and DS are sandy clay. Those from DS are silty (13%) than those from HS (<5.7%). Termite mound materials in DS are moderately plastic, while those in HS are highly plastic. Flexural strength values vary between 2.20 and 2.38 MPa for unfired bricks and between 2.41 and 3.26 MPa for fired bricks, respectively at 1100 and 1050 °C. Compressive strength values are ranged from 2.01 to 3.50 MPa for unfired bricks and 2.44 (1100 °C) to 11.08 MPa (1050 °C), with the best values in the DS area. Water absorption and linear shrinkage values are less than 25% and 5%, respectively, in the studied fired and unfired bricks. The physical and mechanical properties of unfired and fired bricks show that the studied TMS can be used for dense brick manufacturing. Materials from dry savannah exhibit better characteristics as construction materials due to relatively high weathering intensity leading to a spread-out particle size distribution, sintering, which promotes densification by reducing the porosity, and the conversion of metakaolinite into primary mullite upon temperature increase.

## 1 Introduction

Lateritic soils cover around 33% of emerged earth area at the world scale [1]. In Cameroon, they represent near to 67% of the national territory [2]. Their vast field of use is not to be demonstrated. There are well over one hundred documented industrial applications of clay materials [3]. In these lateritic soils, termite mounds are usually found in abundance in the Tropics [4]. Termiteria or termite mounds are built from clay soils mixed with saliva and some secretion of the termite colony. Termite mound soils are used as fertilizer in agriculture, material for brick manufacturing, research of groundwater, and prospection of mineral resources like gold and diamond [5]. Numerous studies focus on termite mound soils as raw materials for construction [[6], [7], [8]]. Millogo et al. [6] show that those from Kofila in Burkina Faso are made up of quartz, kaolinite, and feldspar. Their geotechnical properties are good for building as compared to surrounding clay materials [6,9].

Many authors have studied the characteristics of clay materials for the production of unfired bricks [[10], [11], [12], [13], [14], [15]], fired bricks [[2], [16], [17], [18], [19], [20], [21], [22]] and both types [23]. The manufacturing of unfired brick most probably requires the use of ordinary Portland cement or chemical reagents such as lime or sodium silicates for its stabilization, while fired bricks are energy-intensive and emit carbon dioxide [10,11]. According to Muheise-Araalia and Pavia [15], stabilization with a standard hydraulic-lime mortar improved the durability of unfired bricks but lowered their strength and vapor permeability and did not significantly change their thermal properties. Oti et al. [[11], [12]] demonstrate that the compressive strength, moisture content, rate of water absorption, percentage of void, density, and durability assessment were all within the acceptable engineering standards for clay masonry units. The increase in phosphate binder content improves the performance of the lateritic soil-based unfired bricks [14]. However, cement has the disadvantage of a high cost and contributing 7% of CO_2_ emissions to the atmosphere [24]. For the manufacture of fired bricks, a higher firing temperature and slower firing cycle have a beneficial effect on the physico-mechanical properties of ceramic products while using the same shaping technique [25]. The low mechanical strength of the firing products is correlated with the proportion of Fe_2_O_3_ in lateritic clays [19,21,[26], [27], [28], [29]]. The mullite and hematite peaks are well developed with the increase in firing temperature [30]. By reducing the porosity of the ceramic body and causing the creation of a considerable liquid phase, which entered and separated the nearby pores, sintering promotes the densification of the samples [17,18,31]. The test results have revealed that the compressive strengths of fired lateritic soil bricks at a very low temperature of 550 °C are higher than those of the same bricks stabilized with lime or cement [23]. These works are focused on clayey materials resulting from the weathering under the same climatic conditions and not on the influence of hydromorphic conditions on the characteristics of clay bricks.

In the Obala-Mbandjock area in Centre Cameroon, the geotechnical properties of lateritic gravels used for road construction vary despite being developed on the same rock [32]. Materials from DS present better geotechnical properties than those from HS. These differences in behavior are the result of intensive weathering processes in the DS area due to good drainage, compared to that of HS areas [33]. According to Ndzié Mvindi et al. [32], the lateritic materials of humid savannah are gravels and fine clayey sand, which are generally plastic, while those of dry savannah are coarser and less plastic. These differences in the intensity of weathering processes could also induce differences in the physical and mechanical properties of compressed earth bricks manufactured with raw materials, especially termite mound soils from both contrasting areas. This work aims to characterize termite mound soils from HS and DS areas in Centre Cameroon for their use in fired bricks production and to highlight microclimate influence on their geotechnical characteristics.

## 2 Location of termite nests and experimental methods

**Fig. 1:**
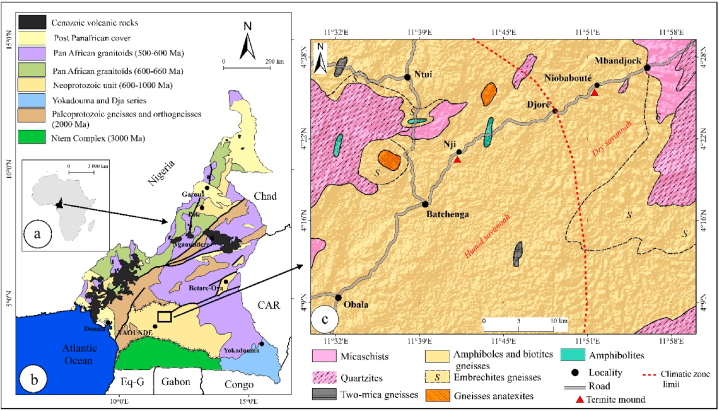
Location map of termite mounds.

**Fig. 2:**
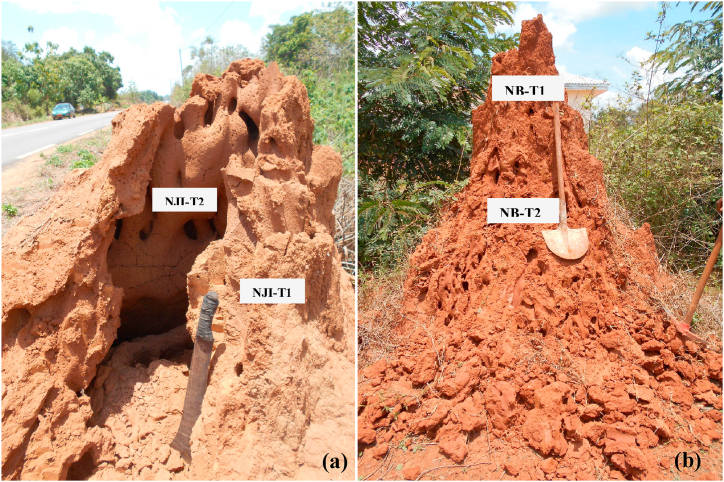
Termite mound from Nji (a) and Niobaboute (b).

Raw materials for compressed earth bricks (CEB) and fired bricks were collected from termite mound soils (TMS) in the Obala-Mbandjock area (Fig. 1), where weathered materials are developed on biotite gneiss. The first termitaria nest is located at NJI in the HS area, while the second is located in Niobaboute in the DS area. For each termite mound, one sample was collected on the external part and another one on the internal part as shown by Fig. 2a and b. The samples of the outer parts were collected after a simple refreshment of the termite mound surface by scraping with a shovel, while the samples of the inner parts were taken from the core of the termite mounds with a pickaxe and shovel. Samples collected at NJI are indexed as NJI-T1 (external) and NJI-T2 (internal). Those collected at Niobaboute are NB-T1 (external) and NB-T2 (internal). The mineralogy of raw materials and fired bricks (900, 1000, and 1100 °C) was determined by powder X-ray diffraction (XRD). The analytical instrument used is a PANalytical X'Pert Pro diffractometer with a monochromator equipped with a cobalt Kα radiation (λ = 1.7890 Å) over a range of 2.5–35° 2θ and a step size of 0.05° 2θ/min at 40 kV and 45 mA. After sample ignition, major elements concentration was determinate using PANalytical Axios Advanced PW 4400 equipment. The particle size distribution of raw materials was obtained by sieving for sizes greater than 75 μm according to the ASTM D6913 (2017) method and by sedimentation for sizes less than 75 μm following the ASTM D422-63 (2016) method.

Atterberg limits (liquid limit, plasticity limit) and plasticity index were measured according to the ASTM D4318 (2017) standard. The determination of the weight content of organic matter was carried out by calcination at a temperature of 450 °C with reference to the XP P94 – 047 method (AFNOR, 1998). For each raw material, eight test briquettes were manufactured using a laboratory hydraulic press. Four of them had parallelepiped shapes (160 mm × 40 mm x 40 mm), while four others were in cubic shapes (40 mm × 40 mm x 40 mm). Based on dry material, 18% water was added to achieve good workability. The parallelepiped samples were pressed at 15 MPa, while the cubic samples were pressed at 62 MPa with a manual laboratory press of 10 tons. Before firing, bricks were dried for seven days in the air and then for 24 h at 105 °C in an oven. A muffle furnace with a maximum operating temperature of 1250 °C was used for the firing. Five firing temperatures were applied to these briquettes: 900, 950, 1000, 1050, and 1100 °C. Before the furnace cooled to room temperature, for each firing cycle, a heating rate of 5 °C/min and a soaking duration of 2 h at the necessary temperature were observed. The color of fired bricks was determined using the Munsell Soil Colors Charts: a brick was placed on a piece of white paper, and their color parameters (hue/value/chroma) were determined by visual comparison with those of standard soils recorded in the Munsell Color Chart under ambient lighting.

The bulk density and apparent porosity have been determined in accordance with the ASTM C20-00 (2015) standard.

Linear shrinkage (LS) was determined using Eq. (1) below (ASTM C531 2000):

(1) $$LS\;\left(\%\right)=\left[\left(L_{0}-L_{1}\right)/L_{0}\right]\times 100$$

where L_0_ is the brick's initial length (mm) after 24 h of drying at 105 °C, and L_1_ is the length (mm) after firing at the specified temperature. Archimede's water displacement method is used to assess water absorption (WA) in accordance with ASTM C20-00 (2015) standard using Eq. (2) below:

(2) $$WA\;\left(\%\right)=\left[(W_{2}-W_{1})/W_{1}\right]\times 100$$

where W_1_ is the mass of the dry-fired brick and W_2_ is the mass of the specimen after a 24 h water-soaked.

**Fig. 3:**
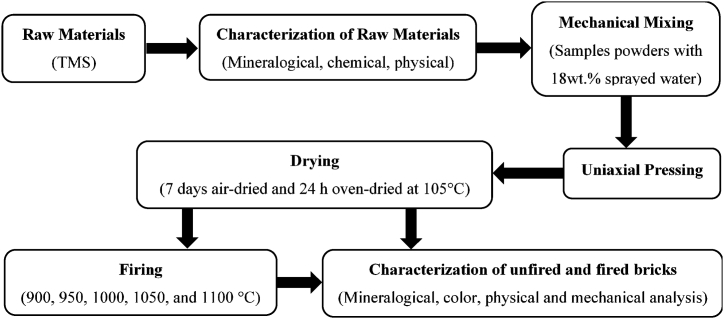
Methodology for production and testing.

Compressive strength (σ_c_) and Flexural strength (σ_f_) values were evaluated by an Impact-Test hydraulic press in the Laboratory of Theoretical and Applied Physical and Analytical Chemistry at the University of Yaounde I (Cameroon) using ASTM C67/C67 M (2018) methods**.** Cubic briquettes were used to measure σ_c_ while parallelepiped ones were used to evaluate σ_f_. The methodology used for the production of unfired and fired bricks and the testing is summarized in Fig. 3.

## 3 Results and discussion

### 3.1 Mineralogical, geochemical, and physical characterization of termite mound materials in dry and humid savannahs

**Fig. 4:**
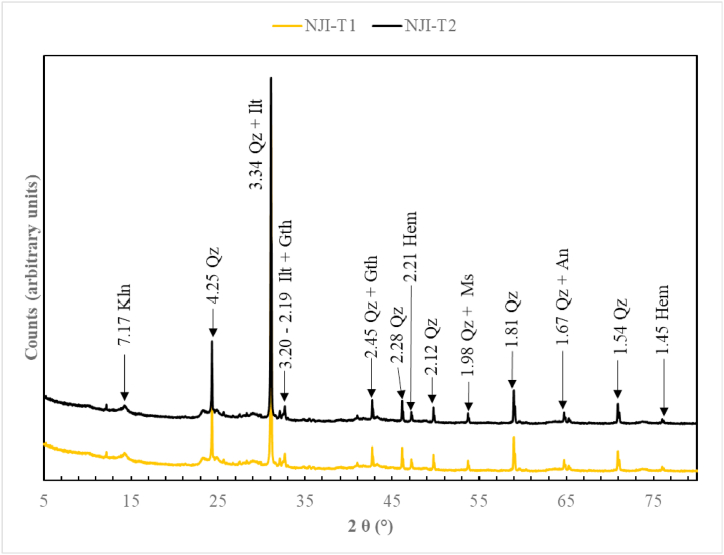
Diffractograms of termitaria materials from NJI. Qz: quartz, Kln: kaolinite, Ilt: illite, Hem: hematite, Gth: goethite; Ms: muscovite, An: anatase.

**Fig. 5:**
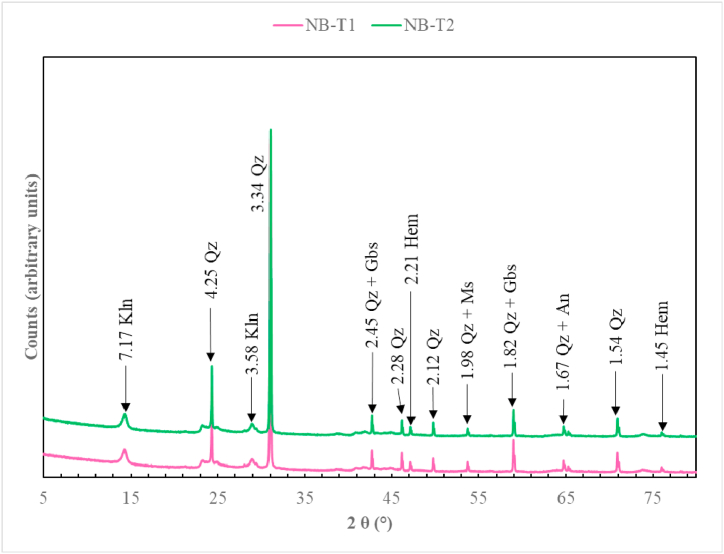
Diffractograms of termitaria materials from Niobaboute. Qz: quartz, Kln: kaolinite, Hem: hematite, Gth: goethite; Ms: muscovite, Gbs: gibbsite, An: anatase.

**Fig. 6:**
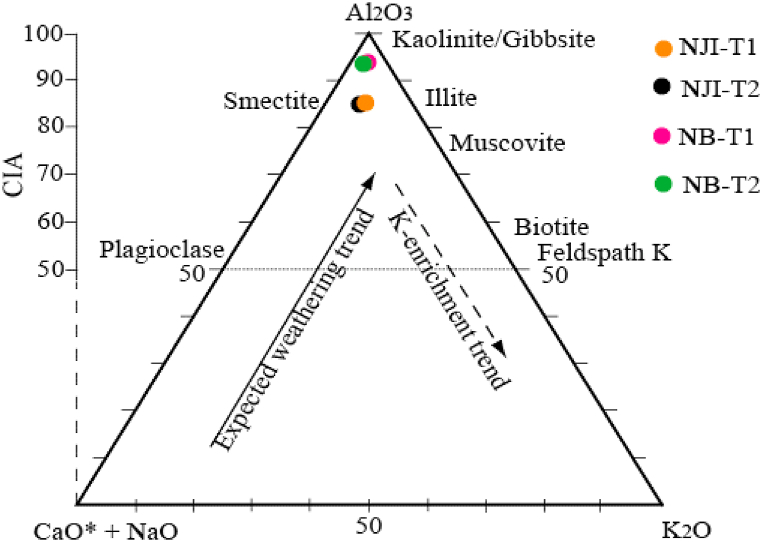
Molar A–CN–K ternary diagram showing the different weathering degrees of the termite mound materials.

Termite mound materials from Nji are made of quartz, muscovite, illite, anatase, kaolinite, hematite, and goethite (Fig. 4). TMS from Niobaboute are mainly composed of quartz, muscovite, anatase, kaolinite, gibbsite, hematite, and goethite (Fig. 5). Hematite is found in few quantities in both area materials. Differences in these mineralogical compositions are due to different levels of weathering, most intensive in DS (Niobaboute) as shown by the ternary A–CN–K molar diagram (Fig. 6). The average thickness of the weathering profile is 12.0 m in DS and 6.8 m in HS [33]. The quartz peaks comparatively high intensity in these materials XRD patterns suggested that this mineral might exist in large amounts [17]. This was supported by the high SiO_2_/Al_2_O_3_ ratios (Table 1), which show that each raw material has weak kaolinite proportions as indicated by the peak of this mineral [34]. Hematite is also found in low quantities considering its peak and may induce a light red coloration of raw material and fired bricks. The yellow color of the HS termite mound (Fig. 2a) is probably due to the presence of goethite while the red color of the DS termitaria is related to the high content of Fe_2_O_3_.

**Table 1:** Major elements composition (wt%) of termitaria materials from Obala – Mbandjock area.

	NJI-T1	NJI-T2	NB-T1	NB-T2
SiO_2_	59.54	58.96	61.79	60.87
TiO_2_	1.02	1.03	1.65	1.66
Al_2_O_3_	16.93	16.93	18.09	18.78
Fe_2_O_3_	9.89	10.33	7.41	7.72
MnO	0.11	0.11	0.07	0.07
MgO	0.52	0.53	0.12	0.12
CaO	0.47	0.49	0.10	0.13
Na_2_O	0.54	0.56	0.06	0.07
K_2_O	1.00	0.98	0.08	0.09
P_2_O_5_	0.08	0.08	0.07	0.07
LOI	9.92	9.98	10.02	10.36
Total	100.02	99.98	99.46	99.94
SiO_2_/Al_2_O_3_	3.52	3.48	3.42	3.24
CIA (%)	85.7	85.5	98.5	98.3

CIA (%) = [Al_2_O_3_/(Al_2_O_3_ + Na_2_O + CaO* + K_2_O)] × 100 in molar ratio.

SiO_2_, Al_2_O_3_, and Fe_2_O_3_ are the main oxides of termite mounds in the studied area (Table 1). This is different in termite mounds from Kofila, where SiO_2_ and Al_2_O_3_ represent more than 95 wt% of the chemical composition [6]. The nature of the parent rock could explain this difference. SiO_2_ contents are high in Nji (54.45–59.54 wt%) and Niobaboute (60.87–62.89 wt%) and are related to the presence of minerals such as quartz, kaolinite, and muscovite. According to Manoharan et al. [18], the durability of bricks is affected by the high quartz content in the raw material. This proportion of quartz makes it possible to obtain bricks with a uniform shape after firing [35]. Al_2_O_3_ contents are moderate (16.93–18.78 wt%), while average Fe_2_O_3_ content (7.41–10.33 wt%) is present in both raw materials. Al_2_O_3_ content is associated with kaolinite and gibbsite, while Fe_2_O_3_ is related to hematite and goethite. The combined SiO_2_ and Al_2_O_3_ percentage is greater than 70% for all raw materials and constitutes a good binder for brick production [36]. According to Nyassa Ohandja et al. [21], the reddish color of the fired bricks is due to the high proportion of Fe_2_O_3_ in the raw materials. These higher iron proportions in HS materials can be explained by a relative accumulation of this oxide in the lower parts of the relief, which confirms the presence of goethite in these materials [37,38]. For both microclimates, the proportions of Fe_2_O_3_ remain higher in the internal parts compared to the external parts. Fe_2_O_3_ present in the outer part of termite mounds is much more mobilized than that of the inner part because of its exposure to meteoric conditions [39,40]. MgO, MnO, CaO, Na_2_O, and K_2_O are in very low proportions in the studied materials. These oxides are quickly evacuated during the weathering of Obala-Mbandjock gneisses [33]. Their higher content in Nji's materials than those from Niobaboute is in accordance with a lower degree of drainage that takes place in humid savannah compared to dry savannah. The low CaO content favors the formation of anorthitic plagioclase during firing [41] and prevents shrinkage of raw brick [18]. Flux oxides (Na_2_O, K_2_O) proportions are very low in studied materials (0.54–1.00 wt%) and may induce poor vitrification and densification [22]. The proportions of LOI vary little in the studied materials. They are slightly lower in the materials of the outer parts (9.92 and 10.02 wt% for NJI-T1 and NB-T1) compared to those of the inner parts (9.98 and 10.36 wt%). These values are related to the dehydroxylation of the clay minerals, organic matter oxidation, and decomposition of hydroxides [31,[42], [43], [44]]. The SiO_2_/Al_2_O_3_ ratio varies between 3.24 (NB-T2) and 3.52 (NJI-T1). These values suggest the presence of phyllosilicate and an excess of silica in the form of quartz, as observed in the mineralogical analysis [23,45].

**Fig. 7:**
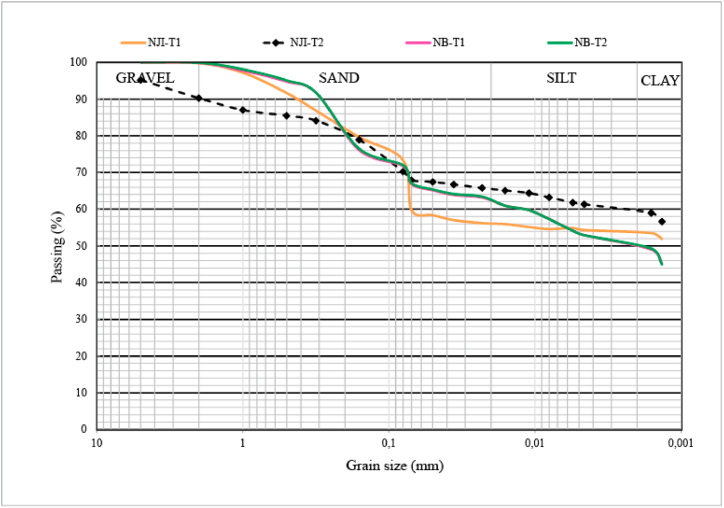
Particle size distribution curves of termite mound materials.

**Fig. 8:**
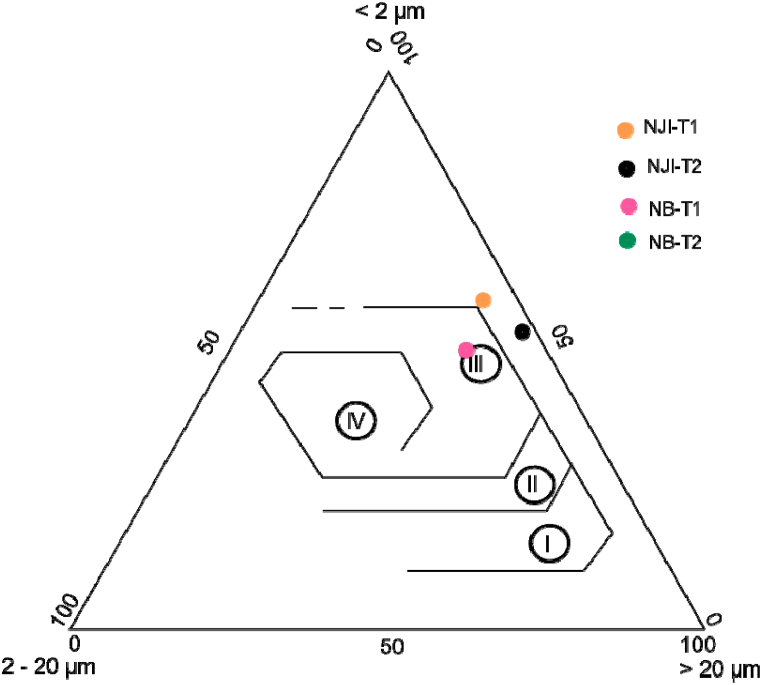
Position of raw materials in Winkler diagram, I: Common bricks; II: Vertically perforated bricks; III: Roofing tiles and masonry bricks; and IV: Hollow products.

The particle size distribution (Table 2) shows that termitaria materials from external and internal parts of Nji (HS) are heterogenous, while those of Niobaboute (DS) are homogenous. Clay (54.0–59.8 wt%) and sand (34.5–44.0 wt%) constitute the main particle size classes of Nji's TMS. At Niobaboute, materials from external and internal parts of the termitaria nest have the same PSD (50.0 wt% of clay, 37.0 wt% of sand, and 13.0 wt% of silt). Silt contents are weak in raw materials from Nji (≤6 wt%); they reach 13 wt% in those from Niobaboute (DS). The homogeneity of Niobaboute's TMS (Fig. 2b) can be explained by the low development of their termitaria compared to those from Nji (Fig. 2a). The higher proportion of clays in the TMS of the HS can be related to the higher thickness of the loose clay horizon in this area compared to the DS exhibit by Kessoum Adamou et al. [33]. Using Shepard's sediment classification [46], TMS are sandy clays. Termite mound materials from Kofila in Burkina Faso are much more sandy (46%) and silty (44%) [6]. Those from Ilorin in Nigeria are silty (40–3%), sandy (37–40%), and clayed (19–21%) [5]. These variations in particle size distribution show that termites use materials as they are available in their area. Most recommendations concerning the contents of the different soil fractions for brick manufacturing are 10–40% for clays, 10–30% for silt, 30–80% for sand, and 0–40% for gravel [47]. Considering these recommendations, studied raw materials have a richer content in clay than required. The particle size distribution curve (Fig. 7) shows that TMS from humid and DS have spread granulometry. The projection of studied materials in the Winkler diagram (Fig. 8) shows that all termite mound materials from Niobaboute in DS can be used in roofing tiles and masonry bricks. Materials from Nji (HS), require the addition of 4–8 wt% of silt for their use at least in roofing tiles and masonry brick production.

**Table 2:** Physical properties of termitaria materials.

		NJI-T1	NJI-T2	NB-T1	NB-T2
PSD (wt%)	Clay	59.8	54.0	50.0	50.0
	Silt	5.7	2.0	13.0	13.0
	Sand	34.5	44.0	37.0	37.0
Atterberg limits (%)	LL	56.7	56.0	46.3	46.4
	PL	38.9	38.4	28.4	28.6
	PI	17.8	17.6	17.9	17.8
OM (%)		1.16	0.85	1.33	0.71

LL-liquid limit; PL-plasticity limit; PI-plasticity index; PSD-particle size distribution; OM-organic matter.

**Fig. 9:**
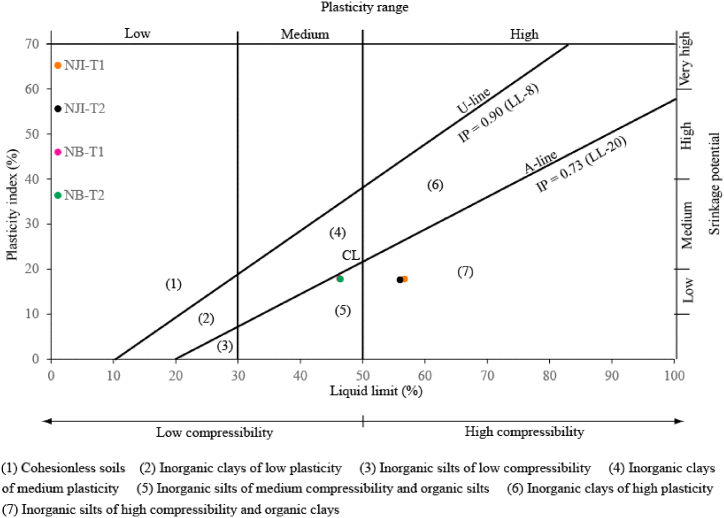
Plasticicy chart of soil samples.

**Fig. 10:**
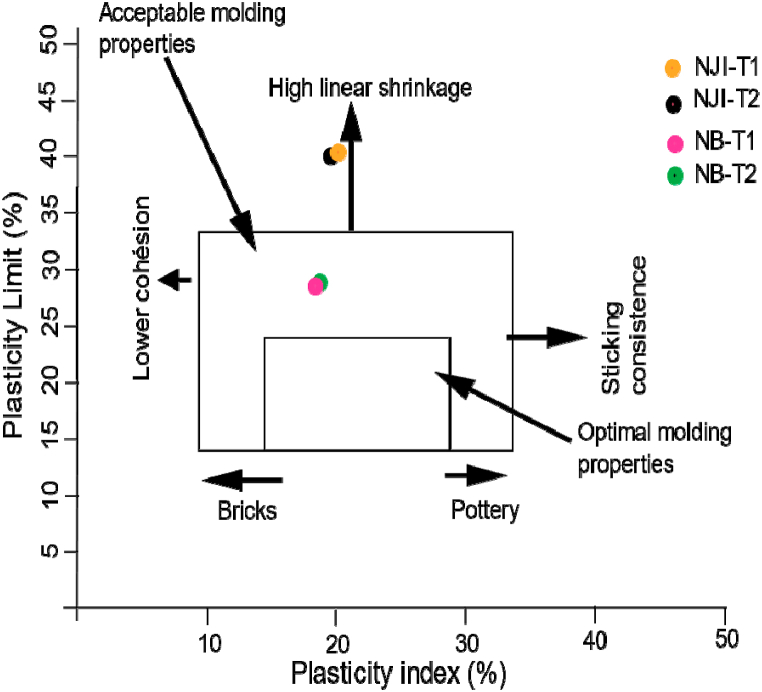
Workability chart of TMS from Nji and Niobaboute.

Concerning Atterberg limits (Table 2), the liquid limit (LL) values vary between 46.3% (NB-T1) and 56.7% (NJI-T1), while the plasticity limit (PL) evolves from 28.4% (NB-T1) to 38.9% (NJI-T1). All the studied materials show low plasticity (PL > 35%) and do not appear cohesive [48]. These values of LL and PL remain higher for materials from the HS area than for those from the DS area. The plasticity index (IP) is framed by values of 17.6% (NJI-T1) and 17.9% (NB-T1), indicating that they had a low degree of plasticity [20]. The Casagrande plasticity chart (Fig. 9) classifies materials from HS as inorganic silts or organic clays with high plasticity, while those from DS are inorganic silts with medium plasticity. Their plasticity is lower than that of TMS from Ika's area in Nigeria [48], in the same range as those from Jawaj and Sene in Ethiopia [49], and higher than that of TMS from N'djamena in Chad [8] and Kofila in Burkina Faso [6]. The termitaria materials from Niobaboute have acceptable molding properties, according to the projection of their plasticity limits and plasticity index in the workability chart (Fig. 10). Materials from NJI are sensitive to high linear shrinkage, certainly due to their high clay contents. According to this workability chart, studied materials require a reduction in their clay content to exhibit the best molding properties.

The organic matter rate (Table 2) varies from 0.71% (NB-T2) to 1.16% (NJI-T1). Due to its contact with vegetation (Fig. 2a and b), the external part of each termite mound presents a higher content of organic matter than the internal one. The studied materials are very poor in organic matter and can be qualified geotechnically as inorganic soils [50]. For brick manufacturing, it is typically advised to use raw materials with an organic matter content of less than 2% [13]. Organic matter is destroyed during firing and can cause cracks when the drying process is made fastly [7]. However, the addition of organic residues to clay bricks from the Afyon region of Turkey improves the compressive strength of unfired bricks in relation to the amount of organic matter [51].

### 3.2 Mineralogy, physical and mechanical properties of compressed earth bricks in dry and humid savannahs

**Fig. 11:**
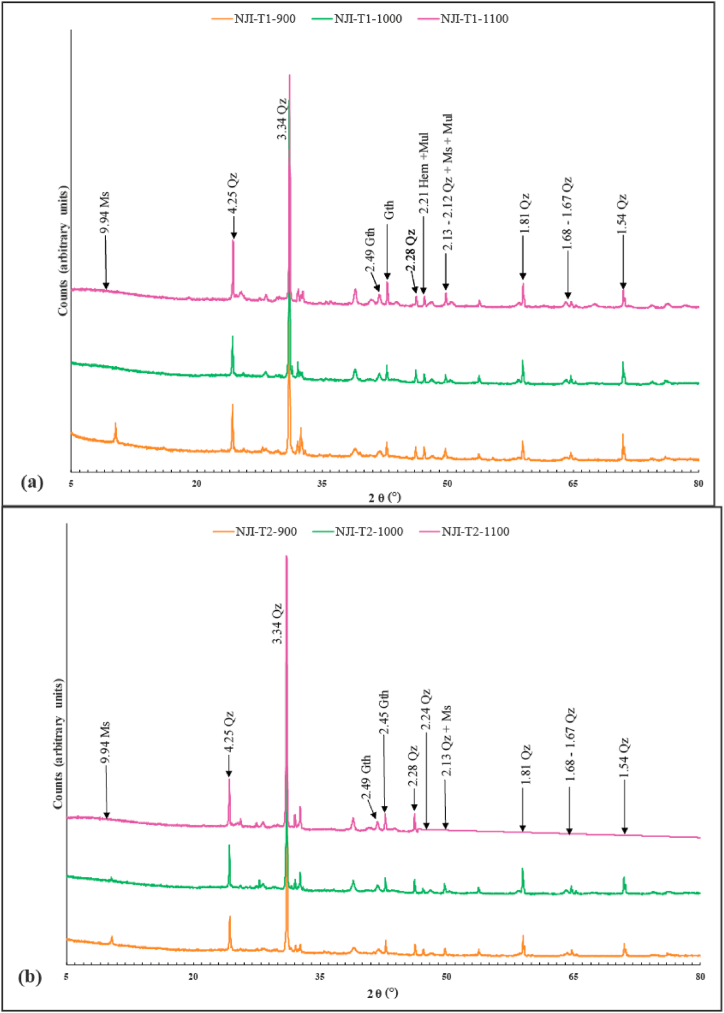
Diffractograms of fired bricks at 900, 1000 and 1100 °C from NJI-T1 (a) and NJI-T2 (b) bricks. Qz: quartz, Ms: muscovite, Ilt: illite, Mul: mullite, Gth: goethite, Gbs: gibbsite.

**Fig. 12:**
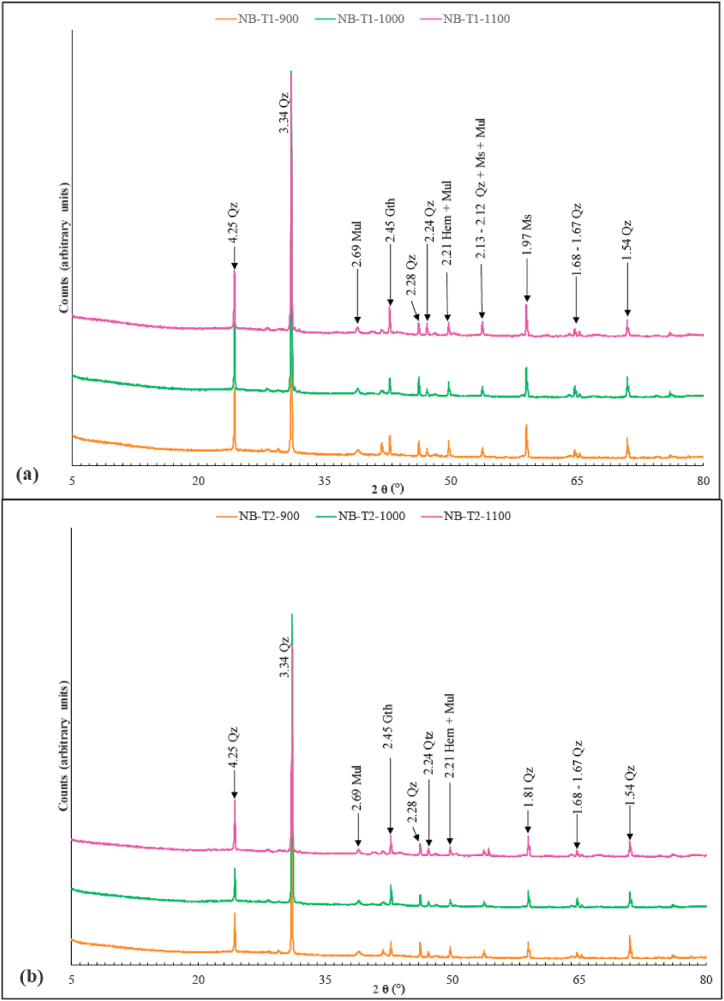
Diffractograms of fired bricks at 900, 1000 and 1100 °C from NB-T1 (a) and NB-T2 (b) bricks. Qz: quartz, Ms: muscovite, Ilt: illite, Mul: mullite, Gth: goethite, Gbs: gibbsite.

Fired bricks from Nji (Fig. 11a and b) and Niobaboute (Fig. 12a and b) are made of quartz, muscovite, mullite, and hematite. Thus, except for kaolinite illite and gibbsite which disappears with firing process [30,52,53], the minerals present in raw materials from Nji and Niobaboute persist after firing until 1100 °C. The gradual transformation of kaolinite into metakaolinite, spinel, and primary mullite is inferred from the disappearance of the peak of this mineral at 900 °C as observed by Lee et al. [54] and McConville and Lee [55].

**Fig. 13:**
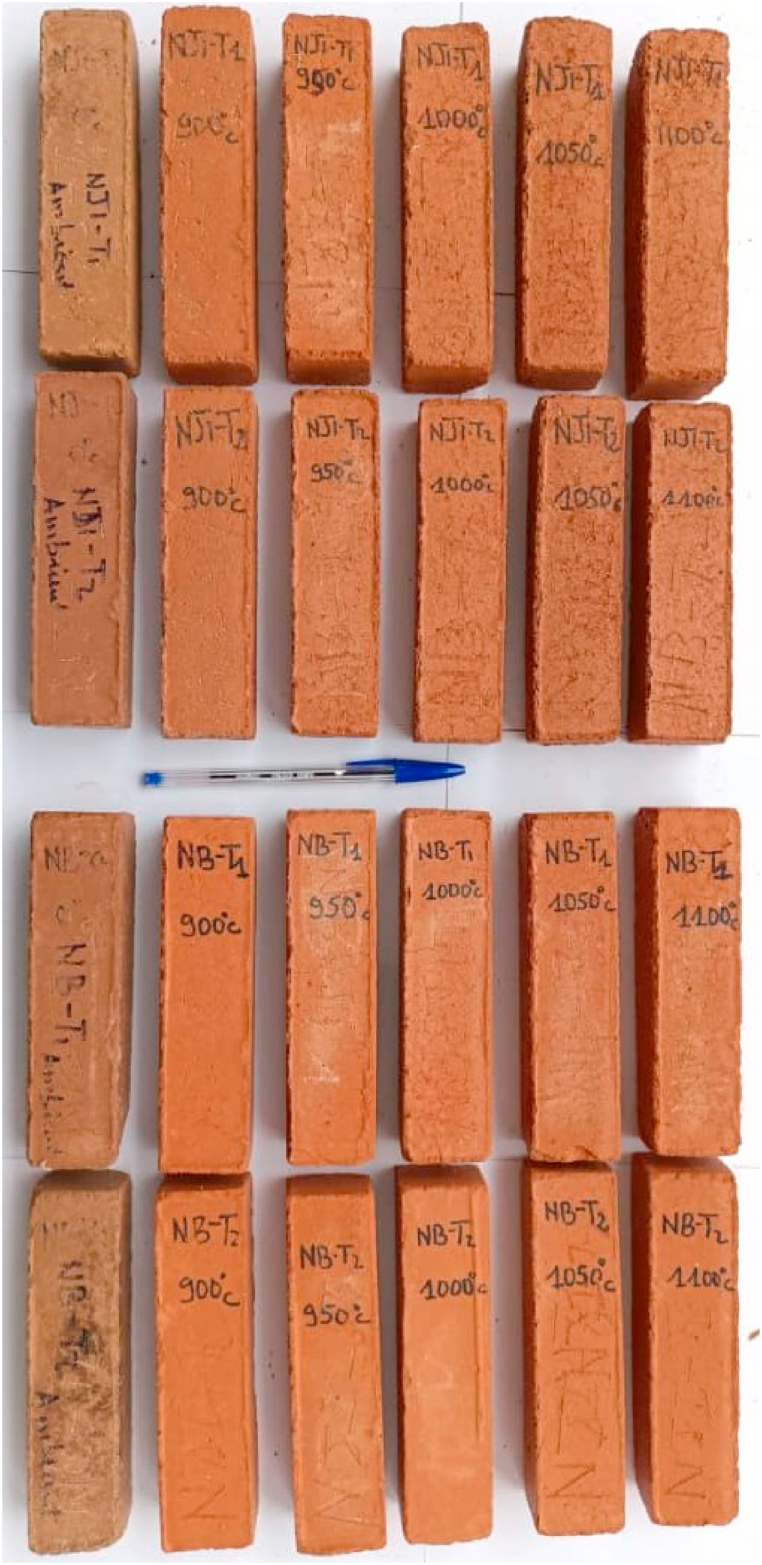
Visual aspect of specimens.

Except for NJI-T1 at ambient temperature which is reddish yellow 5 YR 6/6, CEB from all raw materials and at every firing temperature are light red 2.5 YR 6/6 and 2.5 YR 7/8 (Table 3; Fig. 13). According to Segadaes et al. [56] and Meseguer et al. [57], the presence of iron in the ferric state is responsible for the red colors of fired bricks.

**Table 3:** Munsell code (color) of unfired and fired bricks prepared with termitaria materials from the Obala – Mbandjock area.

Temperature (°C)	NJI-T1	NJI-T2	NB-T1	NB-T2
Ambient	5 YR 6/6	2.5 YR 6/6	2.5 YR 6/6	2.5 YR 6/6
900	2.5 YR 7/8	2.5 YR 7/8	2.5 YR 7/8	2.5 YR 7/8
950	2.5 YR 7/8	2.5 YR 7/8	2.5 YR 7/8	2.5 YR 7/8
1000	2.5 YR 7/8	2.5 YR 7/8	2.5 YR 7/8	2.5 YR 7/8
1150	2.5 YR 6/8	2.5 YR 6/8	2.5 YR 7/8	2.5 YR 7/8
1100	2.5 YR 6/8	2.5 YR 7/8	2.5 YR 7/8	2.5 YR 7/8

**Fig. 14:**
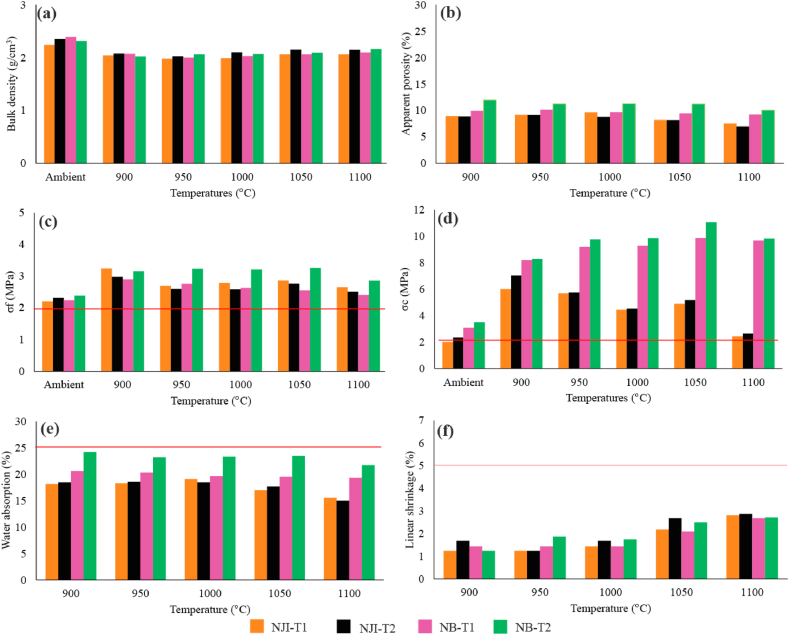
Mechanical and physical properties of bricks. Bulk density (a), apparent porosity (b), flexural (c) and compressive (d) strength of unfired and fired bricks, water absorption, (e) and linear shrinkage (f) of fired bricks.

Bulk density (BD) values are higher for unfired bricks compared to fired bricks (Fig. 14a). They vary between 2.24 g/cm^3^ (NJI-T1) and 2.39 g/cm^3^ (NB-T1) without firing. After firing, BD values overall slightly increase with temperature from 950 °C. They oscillate between 1.98 g/cm^3^ (NJI-T1 at 950 °C) and 2.16 g/cm^3^ (NB-T2 at 1100 °C). The mineralogical changes that occur, such as the formation of mullite, are responsible for this increase in BD with temperature [45,58]. These minor variations were noticed by Ndjigui et al. [22] on the bricks of Kribi, Cameroon, and may be due to the similar mineralogical composition of the samples, in which the main dense phases are quartz and a primary mullite from 1000 °C. Except for NB-T2, apparent porosity values are higher at 950 °C than at 900 °C (Fig. 14b). The lowest AP values are observed at 1100 °C (6.98%–10.06%). There is no fixed maximal porosity for the fired clay bricks [59]. A very high value, however, could pose a problem because the material would be composed of more holes than the solid phase and hence more fragile since this parameter is strongly related to the product's mechanical characteristics.

Flexural strength values (Fig. 14c) oscillate between 2.20 MPa (NJI-T1, ambient temperature) and 3.26 MPa (NB-T2, 1050 °C). After firing, the maximum flexural strength values are obtained at 900 °C for NJI-T1 (3.24 MPa), NJI-T2 (2.98 MPa) and NB-T1 (2.90 MPa) and at 1050 °C for NB-T2 (3.26 MPa). σ_f_ shows its minimum values at 1100 °C for all the studied materials (respectively 2.65, 2.51, 2.41, and 2.86 MPa for NJI-T1, NJI-T2, NB-T1 and NB-T2). Overall, for the same material, this parameter decreases when firing temperature increases, contrary to many previous studies [19,22,34]. This decrease reveals weak densification due to poor mullitization [22,34]. It can also be explained either by the presence of organic matter (>1 wt%) or the contraction of clay which high contents in raw materials develop cracks [7]. Compared to bricks from some lateritic soils from the southern Cameroon plateau, the studied ones display higher flexural strength values than those from Etoug-Ebe, fired until 975 °C [2], from Ayos [19], and those from Nkongbibang-Monatelé [29]. But they are lower than those from Ebebda and Nyondon-Monatele [29]. In the humid savannah, σ_f_ values of external termite mound materials are greater than those of internal parts at all the studied firing temperatures, while in DS the opposite fact is observed. The materials of the internal parts of the termite mound exhibit higher flexural strength in DS as compared to HS. For external-parts materials, the higher σ_f_ values are generally those of humid savannah. Flexural strength for all fired bricks is finally higher than 2 MPa, which is the minimal requirement for standard dense brick manufacturing [16].

For CEB, compressive strength (Fig. 14d) varies between 2.01 MPa (NJI-T1) and 3.50 MPa (NB-T2) at ambient temperature. Globally, σ_C_ increases with firing temperature for materials from DS. In HS, σ_C_ decreases with firing temperature. Bricks from Nji present their maximal compressive strength at 900 °C (6.01 and 7.04 MPa, respectively for NJI-T1 and NJI-T2) while the optimal temperature for those of Niobaboute is 1050 °C for NB-T2 (11.08 MPa) and 1100 °C for NB-T1 (10.47 MPa). Bricks from Niobaboute display the best cohesion and compressive strength regardless of the temperature, as a consequence of their granulometry and a lower iron content than those of Nji [19,[26], [27], [28]]. Compared to bricks from lateritic soils of Etoug-Ebe [2], studied bricks display weak σ_C_ values in the same range of temperatures. This can be explained by the longer dwell times of bricks at the same temperature during firing than in the present study. Mbumbia et al. [2] show that, for the same material and the same firing temperature, materials with a longer dwell time of firing have better compressive strength. Fired bricks manufactured with TMS from Jawaj and Sene in Ethiopia display mean compressive strengths of 14 and 18 MPa [49]. The optimal firing temperature was 700 °C, while temperatures above this caused a decrease in strength. This may be explained either by good particle size distribution or the different mineralogical composition of raw materials between those from Nji and Niobaboute. Except in the case of NJI-T2 at 1100 °C specimens, CEB and fired bricks from both studied areas display values of σ_C_ greater than 2 MPa which is the less internationally required standard for building [47].

Water absorption (WA) values were overall less than 25 wt% and decreased with firing temperatures (Fig. 14e). This parameter has higher values in DS than in HS at all tested firing temperatures. In dry savannah, the materials of the inner part have higher water absorption values than those of the outer part. This fact is only valid in HS at 900, 950, and 1050 °C. The same fact (WA < 25 wt%) was observed for kaolin from Mankon (NW Cameroon) [20] and for alluvial clays from Batouri [34]. This parameter displays a weak decrease as the firing temperature increases. This behavior is probably due to the lack of or very low vitreous phase development [21,34]. This fact is in accordance with the low amounts of fluxing oxides in Nji and Niobaboute TMS materials. Similarly, in bricks made with kaolin from Mankon [20], the highest decrease of water absorption is observed above 1100 °C and it is in line with the enhancement of sintering, which is associated with the start of the vitreous phase formation within the ceramic body [22,34]. Water absorption capacity increases as apparent porosity expands while bulk density and compressive strength decrease [60]. Compared to bricks from some lateritic soils, the studied ones present higher values of water absorption than those from Monatelé and Ebebda [29], lower than those from Etoug-Ebe fired until 975 °C [2], and in the same range as those from Ayos [19]. The combination of this parameter with flexural strength values that are higher than 2 MPa for all studied materials allows using studied TMS for the manufacturing of structural bricks [16].

Linear shrinkage of fired bricks evolves between 1.25 and 2.81% (Fig. 14f). This parameter increases with temperature, though it shows weak variations. These LS values are lower in the external parts, whether in dry or HS at all firing temperatures except at 900 °C for those of dry savannah. Contrary to the workability chart prediction, bricks manufactured with TMS from Nji display low linear shrinkage values despite their high clay contents. This fact could be explained by low CaO content, which favors the formation of anorthitic plagioclase during firing [41] and then prevents shrinkage of raw brick [18]. On the other hand, the absence of swelling clays should also contribute to the same result. Variations in linear shrinkage values are strongly related to those of water absorption. Overall, linear shrinkage values are <5% which is the minimum requirement for traditional ceramic raw materials [6]. Compared to bricks manufactured with TMS from Jawaj and Sene in Ethiopia [49], fired bricks from Nji and Niobaboute display low values of linear shrinkage. In comparison with bricks from some lateritic soils of the surrounding area, the studied ones display very low values of linear shrinkage compared with those from Monatelé and Ebebda [29]. Bricks with lateritic soils from Ayos display lower values of linear shrinkage until 1000 °C but higher above 1050 °C than those studied in the present work [19].

## 4 Conclusions

Termite mound materials from Niobaboute are mainly made of quartz, muscovite, anatase, kaolinite, gibbsite, hematite, and goethite. Those from Nji contain quartz, muscovite, illite, anatase, kaolinite, hematite, and goethite. After firing, kaolinite disappears in favor of primary mullite. They are sandy clays with a spread granulometry, allowing good compaction of CEB. Weathering processes with intensities varying from humid to dry savanna affect the particle size distribution of termitaria raw materials and then command compaction and compressive strength. Whether outside or inside, the plasticity varies slightly for the materials of the same termite mound. However, LL and PL are higher in the HS than in the dry savannah. Considering all the physical and mechanical properties of compressed earth bricks studied, the raw materials of termitaria from the Obala–Mbandjock area are good for building, though they require a reduction of their plasticity. Bricks from DS present better compressive strength than those from humid savanna, and this may be due to the particle size distribution, the sintering, which promotes densification by reducing the porosity, and the conversion of metakaolinite into primary mullite upon temperature increase. Thus, materials from the dry savannah that exhibit better compressive and flexural strength at all the firing temperatures should be recommended over those from the humid area. Studied TMS appear as better raw materials than some lateritic soils, considering the physical and mechanical properties of their bricks.

### Author contribution statement

Jean-Marc Kessoum Adamou: Conceived and designed the experiments; Performed the experiments; Analyzed and interpreted the data; Wrote the paper.

Roger Firmin Donald Ntouala: Conceived and designed the experiments; Analyzed and interpreted the data; Wrote the paper.

Estelle Ndome Effoudou: Analyzed and interpreted the data; Wrote the paper.

Nanga Bineli Marie Thérèse; Arnaud Ngo'o Ze; Gouban Hamadjida: Performed the experiments.

Vincent Laurent Onana: Contributed reagents, materials, analysis tools or data.

## Data availability statement

Data included in article/supp. Material/referenced in article.

## Declaration of competing interest

The authors declare that they have no known competing financial interests or personal relationships that could have appeared to influence the work reported in this paper.
